# Impact of the H274Y Substitution on N1, N4, N5, and N8 Neuraminidase Enzymatic Properties and Expression in Reverse Genetic Influenza A Viruses

**DOI:** 10.3390/v16030388

**Published:** 2024-03-01

**Authors:** Alexandre Gaymard, Caroline Picard, Guilhem Vazzoler, Pascale Massin, Emilie Frobert, Murielle Sabatier, Mendy Barthelemy, Martine Valette, Michèle Ottmann, Jean-Sébastien Casalegno, Bruno Lina, Vanessa Escuret

**Affiliations:** 1 Virpath Unit, CIRI, Inserm U1111, CNRS, UMR5308, ENS Lyon, Université Claude Bernard Lyon 1, F-69372 Lyon, France; 2 Centre National de Référence des Virus des Infections Respiratoires, Groupement Hospitalier Nord, Hospices Civils de Lyon, F-69317 Lyon CEDEX 04, France; 3 Avian and Rabbit Virology Immunology and Parasitology Unit, National Reference Laboratory for Avian Influenza, Anses, Ploufragan-Plouzané-Niort Laboratory, BP53, F-22440 Ploufragan, France; pascale.massin@anses.fr

**Keywords:** influenza A viruses, group-1 neuraminidases, oseltamivir resistance, H274Y-NA substitution, H275Y-NA substitution

## Abstract

The H274Y substitution (N2 numbering) in neuraminidase (NA) N1 confers oseltamivir resistance to A(H1N1) influenza viruses. This resistance has been associated with reduced N1 expression using transfected cells, but the effect of this substitution on the enzymatic properties and on the expression of other group-1-NA subtypes is unknown. The aim of the present study was to evaluate the antiviral resistance, enzymatic properties, and expression of wild-type (WT) and H274Y-substituted NA for each group-1-NA. To this end, viruses with WT or H274Y-substituted NA (N1pdm09 or avian N4, N5 or N8) were generated by reverse genetics, and for each reverse-genetic virus, antiviral susceptibility, NA affinity (Km), and maximum velocity (Vm) were measured. The enzymatic properties were coupled with NA quantification on concentrated reverse genetic viruses using mass spectrometry. The H274Y-NA substitution resulted in highly reduced inhibition by oseltamivir and normal inhibition by zanamivir and laninamivir. This resistance was associated with a reduced affinity for MUNANA substrate and a conserved Vm in all viruses. NA quantification was not significantly different between viruses carrying WT or H274Y-N1, N4 or N8, but was lower for viruses carrying H274Y-N5 compared to those carrying a WT-N5. In conclusion, the H274Y-NA substitution of different group-1-NAs systematically reduced their affinity for MUNANA substrate without a significant impact on NA Vm. The impact of the H274Y-NA substitution on viral NA expression was different according to the studied NA.

## 1. Introduction

Influenza A viruses (FLUAVs) have two major surface glycoproteins: haemagglutinin (HA) and neuraminidase (NA). NA is the target of neuraminidase inhibitors (NAIs). NA is a homotetramer, and its sialidase active site located at the centre of each subunit forms a pocket composed of conserved residues, including catalytic residues that interact directly with the substrate and framework residues that stabilise the active site [1,2,3]. NAs are divided into three phylogenetic groups: group-1 (N1, N4, N5, and N8), group-2 (N2, N3, N6, N7, and N9) from FLUAV, and NA from influenza B viruses [3].

Between 1999 and 2006, oseltamivir-resistant influenza viruses were very rare and reported mainly in immunocompromised patients treated with oseltamivir [4,5]. However, in 2007–2008, oseltamivir-resistant A(H1N1) FLUAV with the H274Y substitution in N1 emerged in Europe [6]. This phenomenon coincided with the emergence of a new seasonal A(H1N1) influenza variant, which may have facilitated the emergence of H274Y-N1 [7,8]. Since the emergence of A(H1N1)pdm09 in 2009, although some clusters of oseltamivir-resistant IV have been described in patients without oseltamivir exposure, the detection of highly reduced inhibition (HRI) FLUAV by NAI remained a rare event [9,10,11]. In addition, the H274Y-NA substitution leading to oseltamivir treatment failure has also been reported in human cases of infection with avian A(H5N1) FLUAV [12,13,14].

A study based on the transfection of 293T cells with plasmids encoding the wild-type (WT) or H274Y-NA of A/Puerto Rico/8/34 (A/PR8) FLUAV, along with flow cytometry to measure NA expression using a monoclonal antibody, showed that the H274Y-NA substitution decreased the amount of NAs at the cell surface. This result was consistent with the lower activity of H274Y-NA measured using the fluorogenic MUNANA (2′(4-methylumbelliferyl)-α-D-N-acetylneuraminic acid) substrate [8]. A study using the same methodology found that the H274Y-N1 substitution of A(H1N1)pdm09 reduced NA activity and cell surface NA expression by 50% [15]. These studies suggested that the reduced expression of H274Y-NA in transfected cells may explain the reduced NA activity observed in viruses; however, to our knowledge, the effect of the H274Y-NA substitution on NA expression at the virion level has not been investigated.

The aim of the present study was to evaluate the effect of the H274Y-NA substitution on each of the group 1 NAs on their antiviral resistance profile, enzymatic properties, and expression in viral particles.

## 2. Materials and Methods

### 2.1. Viruses and Cells

The A/Lyon/969/2009(H1N1) FLUAV was the first A(H1N1)pdm09 FLUAV characterised by the National Influenza Center in Lyon [16]. FLUAV with N4 from A/Turkey/Ontario/6118/68 (H8N4) was kindly provided by the WHO Collaborating Centre of reference and research on influenza, London. The N5 originated from A/duck/Alberta/60/1976 (H12N5) FLUAV (Pubmed accession number AB288335). This N5 was synthetised by Eurofins MWG/Operon and cloned in a pUC 57 vector. The N8 originated from an HPAI A/decoyduck/France/161105a/2016 (H5N8) and kindly provided by the ANSES laboratory in Ploufragan (France).

ATCC-purchased MDCK cells (CCL34) were used for reverse genetics, virus production, and titration. MDCK cells were maintained in serum-free Ultra-MDCK medium (Lonza, Bale, Switzerland) supplemented with 1% L-Glutamine and 2% penicillin–streptomycin (PS) (Lonza). ATCC-purchased 293T cells (CRL-11-268) were only used for reverse genetics. The cells were maintained in DMEM (Lonza) and supplemented with 1% L-Glu, 2% PS, and 10% FBS.

### 2.2. Cloning of Neuraminidases in pHW2000 Plasmid and Mutagenesis

After extracting viral RNAs using the QIAamp viral RNA mini kit from Qiagen, (Hilden, Germany) a two-step RT-PCR was performed to amplify the N1, N4, and N8 gene segments, as previously described [17]. The NA segments were then cloned into an intermediate vector (Zero Blunt TOPO PCR Cloning Kit for Sequencing; Invitrogen, Waltham, MA, USA), except for N5 which was directly synthesised in pUC57. A gel extraction kit from Macherey Nagel (Hoerdt, France) was used for separation and purification. The NA segments were digested, purified, and finally ligated using T4 DNA ligase (NEBiolabs, Waltham, MA, USA) in pHW2000.

Mutations were introduced into NA genes cloned in pHW2000 using the QuickChange II XL site-directed mutagenesis kit (Stratagene, La Jolla, CA, USA) and appropriate primers (available on request) to substitute H275Y for N1, H274Y for N4, H272Y for N5, and H273Y for N8 (all H274Y using N2 numbering). All recombinant plasmids and all recombinant viruses produced were sequenced (Genoscreen, Lille, France) to ensure fidelity to the initial gene segment and/or the presence of the desired mutations. All numbering used in this paper is based on the N2 numbering.

### 2.3. Generation of Viruses by Reverse Genetics

Recombinant viruses were generated using an eight-plasmid DNA transfection system following a previously described method [18]. The A/PuertoRico/8/1934 (H1N1) FLUAV (abbreviated A/PR8) was used as the virus source. Briefly, 293T cells in co-culture with MDCK cells (ratio of 70:30) were transfected by eight cDNA (PB2, PB1, PA, HA, NP, M, NS, and the NA of interest) and cloned in pHW2000 (1 µg per plasmid) in the presence of lipofectamine (Life Technologies, South San Francisco, CA, USA) in OptiMEM medium (Thermo Fisher Scientific, San Francisco, CA, USA) with 0.3% BSA (Sigma, Lezennes, France). At 24 h post-transfection, the medium was changed, and 1 µg/mL of trypsin (Roche, Bale, Switzerland) was added to the culture. The supernatants were harvested 72 h post-transfection. The experiments were conducted in compliance with French legislation.

### 2.4. NA Enzymatic Assays

Oseltamivir carboxylate, zanamivir, and laninamivir were provided by Roche, GlaxoSmithKline (Brentford, UK), and Daiichi-Sankyo (Tokyo, Japan), respectively. The NA activity assay, the enzymatic kinetic analysis, and the fluorometric inhibition assay were performed at 37 °C using a FLUOstar OPTIMA fluorometer (BMG LABTECH, Champigny-sur-marne, France), as described in previous studies [18,19,20], except MES buffer (pH 6.4) was used. The total NA activities were calculated as the quantity of MUNANA substrate (Sigma) degraded to 4-methylumbelliferone (4-Mu) in 1 h per mL of viral suspensions (nmol of 4-Mu/h/mL). Then, a standardised amount of NA activity (10 nmol of 4-Mu/h/mL) was used for the NA inhibition assay and for enzymatic kinetic assay. The inhibitory concentrations (IC_50_) were calculated using GraphPad (Prism) software (v8.0.2). For the enzymatic kinetic assay, the 4-Mu fluorescence was measured every minute for 1 h using MUNANA substrate ranging from 10 to 200 µM. The initial velocity was calculated for each substrate concentration and integrated into a non-linear Michaelis–Menten equation using the MARS program (BMG) to determine the Michaelis–Menten constant (Km; µM) and the maximum velocity (Vm; expressed in arbitrary unit (U.s^−1^)).

### 2.5. Concentration of Viral Supernatants

Each virus production was inoculated at a MOI of 10^−3^ TCID_50_/cell on confluent MDCK cells in three 5-layer multi-flasks (BD Falcon™, Erembodegem, Belgium). At 72 h post-infection, 750 mL of the supernatant was collected and clarified by centrifugation before being concentrated by tangential filtration (vivaflow 50 cassette [100 KDa threshold]; Sartorius, Göttingen, Germany). 60 mL of the concentrated viruses obtained by tangential filtration was concentrated again through a sucrose cushion (25% [*w*/*v*] in NTE buffer (100 mM NaCl, 10 mM Tris-Cl, and 1 mM EDTA, pH = 7.4)) by ultracentrifugation at 160,000× *g* for 2 h at 4 ° C in an optimum L-80 XP ultracentrifuge (Beckman, Brea, CA, USA). The pellets were taken up in an NTE buffer and stored at −80 °C.

### 2.6. Relative Quantification of NA Expression on Purified Virus by Mass Spectrometry

The relative quantification of the NA was performed on the concentrated purified viruses. After the inactivation of concentrated purified viruses in NTE by mixing 1:1 with lysis buffer (8 M urea and complete protease inhibitor cocktail [Sigma-Aldrich, St. Louis, MO, USA] in 100 mM ammonium bicarbonate) and incubation for 30 min at room temperature, the lysate was clarified (15 min at 15,000× *g*), the protein-containing supernatant was collected, and the protein concentration was estimated using the Bradford assay [21]. The equivalent of 35 μg of protein was precipitated by trichloroacetic acid (TCA) at 4 °C overnight. After two washes with acetone, the pellet was taken up in 20 μL 50 mM NaOH, and 80 μL 100 mM triethylammonium bicarbonate (TEAB) was added. The samples were reduced and alkylated (tris-2-(-carboxyethyl)-phosphine [TCEP]/iodoacetic acid [IAA]) then digested with LysC at a ratio of 1/100 for 5 h at 37 °C and then with trypsin at a ratio of 1/100 overnight at 37 °C.

The peptide concentration of the samples was verified. The samples were Tandem Mass Tag (TMT)-marked according to the supplier’s protocol (Thermo Fisher Scientific). All mass-tagging reagents within a set have the same nominal mass (i.e., they are isobaric) and enable multiplex relative quantitation using high-resolution Mass Spectrometry (MS) for samples prepared from cells. The samples were pooled to have the final equivalent of 3 μg of protein, acidified to 5% final formic acid. The pool was then desalted on a C18 spin column. The samples were analysed in triplicate using a high-resolution orbitrap mass spectrometer (Q Exactive HF Biopharma (Thermo Scientific)) in TOP15 HCD mode, R (MS2): 45K. The data were reprocessed using Proteome Discoverer 2.4 software with the Sequest HT search engine against the bank uniprot Canis Lupus data, a contaminant and protein sequence bank of the influenza virus, and filtered at a false positive rate of 1%. Mutant/WT ratios were calculated for each pair of viruses. The quantification data of the three replicates were averaged, and the *p*-value was calculated. We considered an expression difference for a ratio greater than 2 (log2 R > 1) or less than 0.5 (log2 R < −1) and a *p*-value less than 0.05.

### 2.7. Statistical Analysis

The results of the Vm, the Km, and the IC_50_ assays were analysed by a two-tailed Mann–Whitney test using GraphPad (Prism) software (v8.0.2). The results were considered significantly different for a *p* value < 0.05.

## 3. Results

### 3.1. Enzymatic Characterisation of NAs (Vm and Km) and Inhibition Assays (IC_50_)

**Figure 1:**
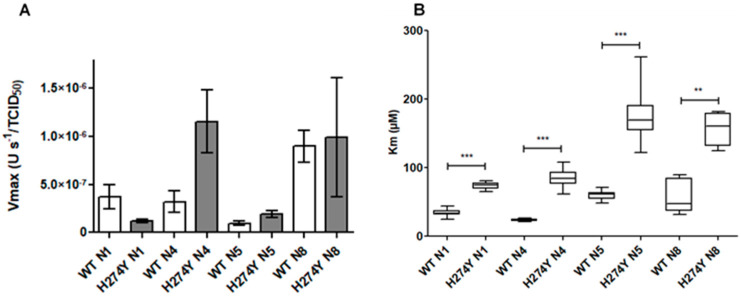
Vm and Km for reverse genetic influenza A viruses bearing WT or H274Y-substituted NA according to the NA subtypes on initial MDCK supernatants. Vm normalised by a viral titre in TCID_50_ are presented on the left part of the figure (**A**), and Km are presented on the right part of the figure (**B**). Data presented are mean values ± standard deviation for normalised Vm, and mean values and minimal and maximal values for Km. The results were analysed by a Mann–Whitney test using Graphpad (Prism) software. The significant differences between the viruses bearing WT and corresponding H274Y-NA were presented (** *p* < 0.01, and *** *p* < 0.001).

The Oseltamivir, zanamivir, and laninamivir IC_50_s for the reverse genetic viruses indicated that the effect of the H274Y substitution was the same for all group 1 NAs (Table 1). Using WHO recommendations for human influenza A virus, the interpretations of inhibition by NAIs were based on the fold increase in IC_50_ values compared to values for susceptible viruses. Normal inhibition (NI) was defined as a less-than-10-fold inhibition, reduced inhibition (RI) as a 10- to 100-fold inhibition, and highly reduced inhibition (HRI) as a greater-than-100-fold inhibition [22]. Viruses carrying the H274Y-N1 substitution had HRI to oseltamivir; there was a 159- to 518-fold increase in oseltamivir IC_50_ depending on the NA subtype. These reverse genetic viruses exhibited NI by these two NAIs (Table 1). The Vm, representing the maximum velocity of the enzyme, normalised by viral titre in TCID_50_, were not significantly different for viruses carrying WT or H274Y-substituted NA (Figure 1A). To compare the effect of the H274Y-NA substitution on the Km between different reverse genetic FLUAVs, we calculated the Km ratio (H274Y-NA Km/corresponding WT NA Km, Table S1). The H274Y-NA substitution induced a significant increase in the mean Km compared to the corresponding WT NA for each FLUAV studied (Figure 1B).

### 3.2. Relative Quantification of NA Expression on Purified Virus by Mass Spectrometry

We analysed whether NAs were differentially expressed in the concentrated viruses bearing the WT or the substituted NA. The H274Y-NA substitution was the only difference between viruses for each pair. After the normalisation of the protein quantity, the digestion and labelling of the peptides, each sample was analysed by MS. This identified 1483 proteins, of which 1465 were from Canis lupus familiaris cells, and 18 were from FLUAV. The peak obtained for each peptide was compared between the WT and mutated virus for each virus pair. The ratios of the arbitrary amounts of peptides used to identify each NA were calculated for each pair of viruses between the sample containing the mutated NA and the sample containing the WT NA. For each pair of virus analysed in triplicate, the mean ratios (mutant/WT) of the NA quantification were 1.025 (*p* = 0.788) for N1, 0.560 (*p* = 8.8 × 10^−5^) for N4, 0.285 (*p* = 2.15 × 10^−7^) for N5, and 1.458 (*p* = 0.013) for N8. Considering an expression difference for a ratio (mutant/WT) greater than 2 (log2 R > 1) or less than 0.5 (log2 R < −1) and a *p*-value less than 0.05, the amount of NA was determined to be significantly different only for viruses carrying N5; there was significantly less N5 detected in virions carrying H274Y-N5. For N1, N4, and N8, the NA quantity was not significantly different between the mutant and the WT viruses. There were no significant differences in the quantity of other viral proteins between the mutant and WT viruses.

## 4. Discussion

This study provides data confirming that H274Y-NA substitution is responsible for the HRI of oseltamivir in FLUAV bearing a group-1 NA with a conserved inhibition of zanamivir or laninamivir. The H274Y-NA substitution induced a systematic and significant increase in Km but impacted NA expression differently according to the NA.

This study presents some limitations. It is of note that we studied only one representative of each group-1 NA, and it is possible that other NAs could behave differently. In addition, as the aim was to compare the impact of the substitution on the different NA subtypes, we used the same A/PR8 background, known to be very permissive, for all the reverse genetic FLUAV produced. However, the genetic background has an impact on the glycoprotein quantities at the virion surface and on viral fitness, and therefore results may be different with another viral background [23].

Regarding the impact of the H274Y-substitution on oseltamivir resistance, the results were similar to those of previous publications investigating other N1 in human or avian FLUAV [10,11,16,24] and other N4, N5, and N8 in reassortant FLUAV [25]. Previous publications have investigated the effect of H274Y-NA substitution on group 2 NAs. For example, a reassortant FLUAV carrying the H274Y-N2 substitution had a mean ± SD oseltamivir IC_50_ of 2.23 ± 0.20 nM (a 9.4-fold increase over WT N2) [26]. This substitution induced only a 2.8-fold increase in oseltamivir IC_50_ for a recombinant N2 protein [27]. Moreover, a reassortant FLUAV bearing an N3, N6, N7, or N9 with H274Y-NA substitution had, respectively, a 12.2-, 1.6-, 17.4-, and 90.3-fold increase in oseltamivir IC_50_ compared to a FLUAV with the corresponding WT NA [25,28]. Zanamivir and laninamivir have a very close structure, explaining the similar low impact of the H274Y substitution on the susceptibility to these NAIs [29]. Structural differences between group-1 and group-2 NAs could explain the differences of the H274Y-NA substitution impact on the susceptibility to oseltamivir according to the NA subtype. Indeed, structural studies have shown that the conformation of E276 is important for an adapted oseltamivir binding in N1 [30], and at position 252, while the Y residue is usually conserved in group-1 NAs, a T or H residue can be found in group-2 NAs [1]. Therefore, the combination of both Y252 and Y274 (Y is a bulky aromatic hydrophobic residue) may prevent the rotation of the E276 residue which is required to accommodate oseltamivir’s hydrophobic chain in N1 [30].

Regarding the impact of the H274Y-NA substitution on enzymatic properties, we observed a systematic and significant increase in the Km of viruses bearing H274Y-NA compared to the WT NA, suggesting that the H274Y-NA substitution decreases the affinity for the MUNANA substrate for all the studied NAs. The Km ratio between H274Y-NA and WT NA was similar to that previously published for N1 of A(H1N1)pdm09 viruses [31] and N1 of human or avian origin [7,24]. 

The H274Y-NA substitution did not impact significantly the Vm for H274Y-group-1-NA compared to WT NA. These results suggest that enzymatic velocity is conserved when the enzyme is saturated by the substrate.

It is difficult to interpret the impact of the H274Y-NA substitution on NA activity alone, motivating our aim to also quantify its impact on NA expression at the virus level. The measured NA activities are theoretically dependent on two factors: the absolute number of NA proteins and their enzymatic capacity towards the MUNANA substrate. In the present study, the H274Y-NA substitution significantly decreased the NA expression for N5 only, but it did not significantly modify NA expression for N1, N4, or N8. These results on N1 seem discordant with previous studies reporting that H274Y-N1 substitution decreased the expression of NA by 50% at the cell surface, explaining the 50% decrease in NA activity [8,15]. However, conversely to these previous publications, we used a different approach, suggesting that observations made after transfection experiments at the cellular level may not be directly transposable in viral particles.

## 5. Conclusions

In conclusion, the H274Y-NA substitution systematically induced an HRI of oseltamivir and decreased affinity for MUNANA substrate for all the group-1 NA studied, but the Vm was not significantly different between the WT and the mutated NA. The impact of the H274Y on NA expression in viral particles seemed different for each studied NA.

## Figures and Tables

**Table 1:** Mean values of oseltamivir, zanamivir, and laninamivir IC_50_ for reverse genetic influenza A viruses with WT or H274Y substituted NA.

Viruses with Different NA ^a^	IC_50_, nM ^b^ (Ratio) ^c^		
	Oseltamivir	Zanamivir	Laninamivir
WT N1	0.30 ± 0.09	0.45 ± 0.16	0.50 ± 0.16
H274Y N1	121.82 ± 33.55 (406) ***	0.53 ± 0.18 (1.18)	1.00 ± 0.38 (2.00) **
WT N4	1.01 ± 0.47	0.94 ± 0.43	0.80 ± 0.55
H274Y N4	160.47 ± 33.26 (159) ***	1.97 ± 0.65 (2.10) ***	4.00 ± 1.00 (5.0) ***
WT N5	0.73 ± 0.27	0.59 ± 0.27	0.41 ± 0.12
H274Y N5	378.01 ± 79.64 (518) ***	1.14 ± 0.34 (1.93) ***	1.88 ± 0.82 (4.59) ***
WT N8	0.30 ± 0.13	0.43 ± 0.17	0.53 ± 0.11
H274Y N8	107.37 ± 41.10 (358) ***	0.90 ± 0.58 (2.09) ***	1.82 ± 0.46 (3.43) **

Data are mean values ± standard deviation for oseltamivir, zanamivir, and laninamir IC_50_ obtained on initial and concentrated MDCK cells supernatants. Results were compared using a two-tailed Mann–Whitney test using GraphPad (Prism) software. ** *p* < 0.01 and *** *p* < 0.001 for the test against the corresponding WT NA. ^a^ Names of the NA of reverse genetic FLUAV with the PB1, PB2, PA, HA, NP, M, and NS segments from A/Puerto Rico/8/34 (H1N1) and NA segment from different origins. ^b^ IC_50_ were determined using fluorometric assays. ^c^ Numbers in parentheses correspond to the fold differences in the IC_50_ between reverse genetic FLUAV with a substituted NA versus the corresponding FLUAV with WT NA. Interpretations of FLUAV inhibition by NAIs are based on fold increases in IC_50_ values compared to values for susceptible viruses: normal inhibition was defined as a <10-fold inhibition, reduced inhibition as a 10- to 100-fold inhibition, and highly reduced inhibition as a >100-fold inhibition (bold).

## Data Availability

The data presented in this study are available on request from the corresponding author.

## Supplementary Materials

The following supporting information can be downloaded at: https://www.mdpi.com/article/10.3390/v16030388/s1, Table S1: Mean values of Km, Vm, activity, and IC50 for concentrated reassortant influenza A viruses.
