# Supplementary material for: Impact of the H274Y Substitution on N1, N4, N5, and N8 Neuraminidase Enzymatic Properties and Expression in Reverse Genetic Influenza A Viruses
Source: Viruses. 2024 Mar 1;16(3):388. doi: 10.3390/v16030388 (PMC10975200; doi:10.3390/v16030388)
Supplement: Supplementary file 1 [file viruses-16-00388-s001.zip › viruses-2811321-supplementary.pdf]

Supplementary Table S1

Table S1: Mean values of Km, Vm, activity, and IC_{50} for concentrated reassortant influenza A viruses

Virus<br>RG PR8 -NA	Km (µM) (Ratio)	Vmax (s^{-1})	Activity (nmol of 4Mu h^{-1} mL^{-1})	IC_{50} (nM) (Ratio)			Viral Titer
				oseltamivir	zanamivir	laninamivir	$log_{10}[TCID_{50}]$/mL
N1 WT (P5 vivaflow)	23.00 ± 1.00	15.67 ± 0.58	325 ± 69	0.38 ± 0.02	0.43 ± 0.07	0.41 ± 0.05	9.30
N1 WT (P5 ultracentrifugation)	18.00	13.00	4740	0.46	0.56	0.56	10.63 / 10.30
N1 H274Y (P5 vivaflow)	52.33 ± 6.11 (2.28)	18.33 ± 1.15 (1.17)	124 ± 45 (0.38)	**148.57 ± 14.38 (391)**	0.43 ± 0.07 (1.00)	0.68 ± 0.15 (1.66)	9.30
N1 H274Y (P5 ultracentrifugation)	34.00 (1.89)	22.00 (1.69)	2960 (0.62)	**162.30 (353)**	0.53 (0.95)	0.90 (1.61)	9.92 / 10.30
N4 WT (P6 vivaflow)	17.33 ± 2.08	18.00 ± 0.00	207 ± 56	1.27 ± 0.10	0.76 ± 0.25	0.46 ± 0.17	8.97
N4 WT (P6 ultracentrifugation)	11.00	11.00	4300	0.79	0.63	0.63	9.53 / 10.55
N4 H274Y (P6 vivaflow)	59.17 ± 9.75 (3.41)	16.00 ± 1.00 (0.89)	112 ± 23 (0.54)	**186.97 ± 17.92 (147)**	2.01 ± 0.71 (2.64)	3.77 ± 0.62 (8.20)	9.63
N4 H274Y(P6 ultracentrifugation)	33.00 (3)	18.00 (1.64)	3060 (0.71)	**191.30 (242)**	1.22 (1.94)	3.56 (5.65)	10.63 / 10.80
N5 WT (P5 vivaflow)	46.00 ± 6.24	16.00 ± 1.00	748 ± 182	0.97 ± 0.09	0.42 ± 0.10	0.36 ± 0.04	9.80
N5 WT (P5 ultracentrifugation)	42.00	11.00	11360	0.74	0.70	0.39	10.63 / 10.39
N5 H274Y (P7 vivaflow)	132.33 ± 15.89 (2.88)	18.33 ± 1.15 (1.15)	128 ± 24 (0.17)	**455.20 ± 79.93 (469)**	0.95 ± 0.16 (2.26)	1.12 ± 0.29 (3.11)	8.30
N5 H274Y (P7 ultracentrifugation)	118.00 (2.81)	19.00 (1.73)	2500 (0.22)	**408.00 (551)**	1.27 (1.81)	1.18 (3.03)	10.80 / 10.39
N8 WT (P7 vivaflow)	23.00 ± 1.73	16.33 ± 0.58	174 ± 66	0.49 ± 0.06	0.47 ± 0.10	0.55 ± 0.16	8.30
N8 WT (P7 ultracentrifugation)	17.00	15.00	2680	0.35	0.55	0.53	9.97 / 9.89
N8 H274Y (P7 vivaflow)	88.00 ± 12.73 (3.83)	22.25 ± 0.96 (1.36)	211 ± 46 (1.21)	**168.50 ± 7.83 (344)**	1.51 ± 1.07 (3.21)	2.08 ± 0.42 (3.78)	9.30
N8 H274Y (P7 ultracentrifugation)	79.00 (4.65)	16.00 (1.07)	2780 (1.04)	**131.70 (376)**	0.94 (1.71)	1.87 (3.53)	10.80 / 10.39

For each reassortant IAV bearing a WT or substituted NA, enzymatic NA characteristics and IC_{50} were measured on MDCK cells supernatants concentrated by tangential filtration (three values) and ultracentrifugation (one value). Viral titres in $TCID_{50}$ are also mentionned. For supernatants concentrated by tangential filtration, before ultracentrifugation, data are presented as mean values ± standard deviation of Km, activity or IC_{50} values obtained for each sort of reassortant IAV. For Km and NA activity, results were analysed by a two-tailed Mann-Whitney test using GraphPad (Prism) software. The difference was significant only for the test comparing Km value between WT NA and H274Y-N8 (* $P<0.05$).

[a] Names of the NA of reassortant IAV with the PB1, PB2, PA, HA, NP, M and NS segments from A/Puerto Rico/8/34 (H1N1) and NA segment from different origins.

[b] Km were determined using fluorometric assays and the MUNANA substrate, as described in [18].

[c] Numbers in parentheses correspond to the fold differences in the Km between reassortant IAV with substituted NA versus the corresponding IAV with WT NA. The Km represents the affinity of the NA for the MUNANA substrate. The lower the Km, the higher the affinity of the NA for its substrate is.

[d] IC_{50} were determined using fluorometric assays, as described in Gaymard et al., 2016.

[e] Numbers in parentheses correspond to the fold differences in the IC_{50} between reassortant IAV with a H274Y-NA versus the corresponding IAV with WT NA. Interpretations of IAV inhibition by NAIs are based on fold increases in IC_{50} values compared to values for susceptible viruses: normal inhibition was defined as <10-fold inhibition; reduced inhibition, as 10 to 100-fold inhibition; and highly reduced inhibition, as >100-fold inhibition (bold characters).

Abbreviation: Km, Michaelis-Menten constant. Vm, maximum velocity. IAV, influenza A virus. NA, neuraminidase. NAIs, Neuraminidase inhibitors. IC_{50}, NAIs concentration able to inhibit 50% of the NA activity. WT, wild-type.
